# Comparative analysis of ascorbate peroxidases (APXs) from selected plants with a special focus on *Oryza sativa* employing public databases

**DOI:** 10.1371/journal.pone.0226543

**Published:** 2019-12-19

**Authors:** Baomei Wu, Binbin Wang

**Affiliations:** 1 International Center for Plant Molecular Genetics, School of Life Science, Shanxi Normal University, Linfen, PR China; 2 School of Chemical Engineering and Technology, Tianjin University, Tianjin, PR China; University of Innsbruck, AUSTRIA

## Abstract

Reactive oxygen species (ROS) are produced by plants. Hydrogen peroxide (H_2_O_2_) is one important component of ROS and able to modulate plant growth and development at low level and damage plant cells at high concentrations. Ascorbate peroxidase (APX) shows high affinity towards H_2_O_2_ and plays vital roles in H_2_O_2_-scavenging. In order to explore the differences of APXs from selected plant species, bioinformatics methods and public databases were used to evaluate the physicochemical properties, conserved motifs, potential modifications and cis-elements in all the APXs, and protein-protein network and expression profiles of rice APXs. The results suggested that APXs in the selected plant species showed high evolutionary conservation and were able to divide into seven groups, group I to VII. Members in the groups contained abundant phosphorylation sites. Interestingly, group I and VII had only PKC site. Additionally, promoters of the APXs contained abundant stress-related cis-elements. APXs in rice plant were able to interact with dehydroascorbate reductase 2. The eight APXs expressed differently in root, leaf, panicle, anther, pistil and seed. Drought, Pi-free, Cd and *Xanthomonas oryzae pv*. *oryzicola* B8-12 treatments were able to significantly alter the expression profiles of rice APXs. This study increases our knowledge to further explore functions and mechanisms of APXs and also guides their applications.

## Introduction

Plants are able to produce reactive oxygen species (ROS) during growth and development, abiotic and biotic stresses. ROS mainly contains singlet oxygen (^1^O_2_), superoxide radical (O_2_^.−^), hydrogen peroxide (H_2_O_2_) and hydroxyl radical (OH^.^) [1]. Among the major ROS, H_2_O_2_ is the only molecule able to cross membrane via plasma membrane aquaporins and, therefore, to move from production sites to the distant with water [2, 3]. In plant cells, the rate of H_2_O_2_ production is the highest in peroxisomes (10,000 nmol/m^2^s), followed by chloroplast (4030 nmol/m^2^s) and mitochondria (< 398 nmol/m^2^s) [4]. Meanwhile H_2_O_2_ is more stable with a half-life of 1 ms, compared with other major ROS [5]. Previous reports have shown that this molecule plays dual roles in plant metabolism [6]. H_2_O_2_ acts as signaling molecule to regulate plant growth and response to stimulus at low concentrations. On the other hand, high levels of H_2_O_2_ result in plant oxidative stress and damage biological macromolecules [7–9]. In order to maintain H_2_O_2_ homeostasis to protect cells from oxidative damage, plants have developed antioxidant enzymes including ascorbate peroxidase (APX), glutathione peroxidase (GPX), catalase (CAT), peroxiredoxins (PRXs) and 2-Cys PRXs to degrade this molecule, several reports also suggested that ascorbic acid (ASC), glutathione (GSH), carotenoids, flavonoids, anthocyanins, α-tocopherol were able to assist the above-mentioned enzymes or directly scavenge H_2_O_2_ [10–12]. Among the enzymes or proteins, APX may play a specific role in H_2_O_2_-scavenging due to its high affinity towards hydrogen peroxide [13].

APX is comprised of different isoenzymes, which are encoded by a multi-gene family and found in many compartments of cell. This enzyme catalyzes the conversion of H_2_O_2_ into H_2_O with ASC as electron donor in ascorbate-glutathione (ASH-GSH) and water-water cycles [14]. Different isoenzymes exhibit different kinetic properties like catalytic rate, optimal pH, stability and molecular weight. APX is assigned to class I of plant superfamily in heme peroxidases [15]. APX genes knock-down or -out in plants result in alteration in growth, physiology and antioxidant metabolism, indicating these enzymes involvement in the plant growth and development [16]. Previous studies showed that functional deficiency of rice APX1 or APX2 resulted in alteration of plant architecture [17], even worse APX2 knock-out mutant reduced fertility [18]. While the rice mutants with double silenced the two APXs exhibited normal phenotype [17]. Studies also suggested that cytosolic APX1 in Arabidopsis played vital roles in protection of chloroplast functions [19], although several APXs were found in the organelle [20]. Consequently, the detailed ROS-scavenging mechanisms and relationships with growth and development of APXs are still unknown.

Under abiotic conditions such as salt, cold, heat and high light, APXs expression profiles and activity could be differentially regulated [14, 21]. When APX genes are overexpressed, the transgenic plants show significant salinity or oxidative tolerance [22–26]. Intriguingly, double silenced for cytosolic APXs in the rice mutant resulted in up-regulation of peroxidases, which made the mutants able to cope with salt, heat, high light and methyl viologen stresses like the non-transformed plants [17]. Meanwhile the same mutant exhibited higher tolerance to aluminum toxic than the wild type rice plants [27]. These studies suggested that APX also involved in several stresses. Studies have shown that APX in the *Arabidopsis* and *Citrus aurantium* is identified as a potential target of tyrosine nitration [28, 29], and NO is able to modulate its activity in different ways [30–32]. Proteomic analysis has been certificated that tyrosine nitration and *S*-nitrosylation could definitely modulate APX activity [33, 34].

In this study, *Chlamydomonas reinhardtii*, *Physcomitrella patens*, *Arabidopsis thaliana*, *Oryza sativa* and *Populus trichocarpa* were selected from Charophyta and Embryophyta. Protein sequences of APXs in the five plant species were downloaded from JGI database. The sequences characterization, evolutionary relationships and potential modification sites in the five species with special focus on *Oryza sativa* and its protein and protein networks and expression profiles of APXs were explored.

## Materials and methods

### Retrieval of APX proteins

APX protein sequences (S1 Text) of *Chlamydomonas reinhardtii*, *Physcomitrella patens*, *Arabidopsis thaliana*, *Oryza sativa* and *Populus trichocarpa* were retrieved from JGI database (https://genome.jgi.doe.gov/portal/) according to annotation and homologs analysis. Subsequently, the obtained sequences were conducted to Hidden Markov Model (HMM) search to confirm the domain families [35]. Species selection in this study obeyed the regularity that they could represent the protists, lower plants, monocots and dicots.

### Sequence analysis of APXs

Molecular weight (Mw), isoelectric point (*pI*) and GRAVY (grand average of hydropathy) of the obtained APXs were investigated by ProtParam tool of Expasy [36]. Subcellular localization (Sub-localization) was predicted by CELLO [37] and WoLF PSORT [38] softwares. Conserved motif structure of the APXs was exploited using the MEME (Multiple Em for Motif Elicitation) software (http://meme-suite.org/) with the following parameters: number of motifs (1–15), motif width of (5–50) [39]. TBtools [40] was used to rebuild the motif maps with the MEME results. While NetPhos 3.1 software [41] was employed to predict the potential phosphorylation sites with scores higher than 0.75 at serine, threonine and tyrosine residues. S-Nitrosylation and S-Palmitoylation sites were analyzed using GPS-SNO 1.0 [42] and CSS-Palm 4.0 [43] softwares with medium threshold, respectively. Additionally, N-Myristoylation, S-Farnesylation and S-Geranylgeranylation sites were detected by GPS-Lipid 1.0 software [44] with medium threshold. Prediction of three-dimensional models was implemented by Swiss-Model software with similar protein models [45]. The models of OsAPX1 and 2, OsAPX3 and 4, OsAPX5, 6, 7 and 8 were produced from PDB 5jqr, 1apx and 1iyn, respectively.

### Phylogenetic analysis of APXs

APX sequences were aligned by ClustalW [46] and the phylogenetic tree was constructed by MEGA 6 [47] with the Neighbor-Joining method for 1500 bootstraps.

### Prediction of potential cis-regulatory elements

Genomic sequences of length 1500 bp upstream to the start codon from *Chlamydomonas reinhardtii*, *Physcomitrella patens*, *Arabidopsis thaliana*, *Oryza sativa* and *Populus trichocarpa* were downloaded from JGI database to predict the putative cis-regulatory elements using PLACE software [48]. The figures were drawn by Microsoft Excel 2010.

### Analysis of interaction network

APXs from *Oryza sativa* were selected and predicted the putative interaction partners with the STRING software [49] and the interaction network was rebuilt and generated by the cytoscape software 3.7.1 [50].

### Expression pattern analysis

The expression data of eight rice APXs were retrieved from rice expression database [51] of IC4R (Information Commons for Rice, http://ic4r.org). Raw data of root, leaf and panicle were from SRP039045, raw data of anther, pistil and seed were from SRP047482, these expression data were calculated by log_10_ (expression value+1). Raw data of 10-day rice seedlings under Cd treatment, 35-day rice plant under Pi-free condition, 45-day rice leaf under drought stress and 16-day old leaf with *Xanthomonas oryzae pv*. *oryzicola* B8-12 infection were from DRP001141, SRP028766, SRP052306 and SRP056884, respectively. During the data analysis, two-tailed student *t* test was used to compare the significance of differences between control and treatment groups.

## Results and discussion

### Retrieval of APX proteins

A total of 36 APX protein sequences, four from *Chlamydomonas reinhardtii*, five from *Physcomitrella patens*, eight from *Arabidopsis thaliana*, eight from *Oryza sativa* and eleven from *Populus trichocarpa*, were retrieved from Phytozome of Joint Genome Institute (JGI). The smallest protein was PtAPX2 from *Populus trichocarpa* with 96 amino acids (aa) among the 36 enzymes, while the largest one was 478 aa from *Oryza sativa* in length (Table 1). The theoretical *pI* ranged from 5.18 to 9.23 and Mw from 10,226.73 to 51,187.63 Da. Additionally, most of the proteins were hydrophilic except for CreAPX2 and CreAPX4 from *Chlamydomonas reinhardtii* and PtAPX2 from *Populus trichocarpa*.

**Table 1:** The basic properties of the 36 proteins.

Species name	Phytozome gene ID	Common name	NO. of amino acid	PI/MW (Da)	GRAVY	Instability index
*Chlamydomonas reinhardtii*	Cre02.g087700	CreAPX1	327	8.67/35663.07	-0.54	41.01
	Cre05.g233900	CreAPX4	347	9.23/36491.76	0.061	41.66
	Cre06.g285150	CreAPX2	337	8.95/35111.29	0.019	44.11
	Cre09.g401886	L-ascorbate peroxidase, heme-containing (CreAPX-heme)	372	8.63/39449.77	-0.169	33.86
*Physcomitrella patens*	Pp3c1_26270	PpAPX3	300	7.01/32672.06	-0.294	35.64
	Pp3c1_40650	PpAPX-S	440	8.11/48253.56	-0.453	45.94
	Pp3c17_7560	PpAPX6-related	357	6.15/38474.81	-0.183	50.38
	Pp3c20_2050	PpAPX2 (PpAPX2.1)	250	5.66/27651.48	-0.364	36.7
	Pp3c20_2100	PpAPX2 (PpAPX2.2)	250	5.53/27759.61	-0.354	36.32
*Arabidopsis thaliana*	AT1G07890	AtAPX1	250	5.72/27561.22	-0.385	33.87
	AT1G77490	AtTAPX	426	6.81/46092.30	-0.284	42.93
	AT3G09640	AtAPX2	251	5.87/28006.04	-0.371	36
	AT4G08390	AtSAPX	372	8.31/40407.32	-0.481	51.66
	AT4G09010	AtAPX4	349	8.59/37933.97	-0.294	35.72
	AT4G32320	AtAPX6	329	8.99/36239.74	-0.184	39.45
	AT4G35000	AtAPX3	287	6.47/31571.86	-0.365	39.4
	AT4G35970	AtAPX5	279	8.80/30895.31	-0.404	33.84
*Oryza sativa*	LOC_Os02g34810	OsAPX8	478	5.36/51187.63	-0.472	53.76
	LOC_Os03g17690	OsAPX1	250	5.42/27155.74	-0.344	42.94
	LOC_Os04g14680	OsAPX3	291	8.25/32047.56	-0.369	45.56
	LOC_Os04g35520	OsAPX7	359	8.76/38325.30	-0.401	42.11
	LOC_Os07g49400	OsAPX2	251	5.18/27117.56	-0.326	39.73
	LOC_Os08g43560	OsAPX4	291	7.74/31738.04	-0.297	34.55
	LOC_Os12g07820	OsAPX6	309	6.72/33501.91	-0.423	52.49
	LOC_Os12g07830	OsAPX5	320	5.83/34759.32	-0.362	52.2
*Populus trichocarpa*	Potri.002G081900	PtAPX-S.1	377	8.68/41003.36	-0.491	50.95
	Potri.004G174500	PtAPX3	286	6.67/31551.92	-0.344	40.95
	Potri.005G112200	PtAPX5	287	7.06/31509.84	-0.336	40.57
	Potri.005G161900	PtAPX-TL29	347	7.59/37842.99	-0.27	46.58
	Potri.005G179200	PtAPX-S.2	467	9.06/51109.32	-0.408	47.74
	Potri.006G089000	PtAPX2	96	5.40/10226.73	0.255	27.56
	Potri.006G132200	PtAPX1.2	249	5.27/27452.91	-0.45	34.13
	Potri.006G254500	PtAPX6 related	337	8.44/36786.03	-0.267	39.94
	Potri.009G015400	PtAPX.3	249	5.53/27318.89	-0.395	34.63
	Potri.009G134100	PtAPX5-like	286	7.06/31444.81	-0.322	34.78
	Potri.016G084800	PtAPX1.1	250	5.48/27577.22	-0.44	32.31

### Phylogenetic analysis of APXs

**Fig 1:**
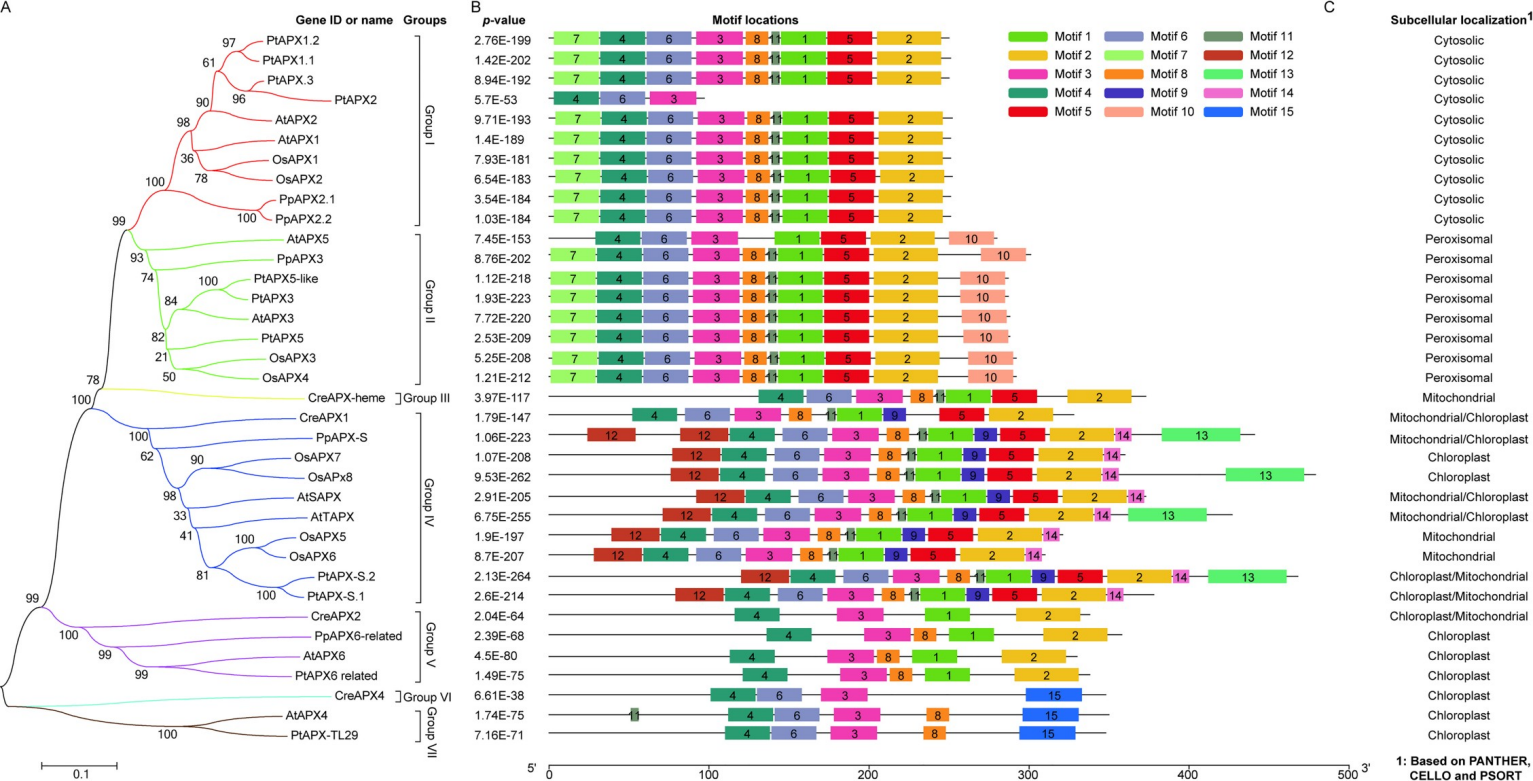
Phylogenetic analysis of APXs from *Chlamydomonas reinhardtii*, *Physcomitrella patens*, *Arabidopsis thaliana*, *Oryza sativa* and *Populus trichocarpa*. A: phylogenetic tree. B: conserved motifs in the 36 APXs. C: Sub-localization of the 36 APXs.

In order to analysis the evolutionary relationship of the 36 proteins, phylogenetic tree was constructed by MEGA 6 software based on neighbor-joining (NJ) method with 1500 bootstraps. Fig 1A showed that the 36 APX proteins were mainly divided into seven groups. Among the seven groups, group III and VI had only one member, respectively. Interestingly, both the two proteins were from *Chlamydomonas reinhardtii*. Group VII had two members, AtAPX4 and PtAPX-TL29, and was, respectively, from *Arabidopsis thaliana* and *Populus trichocarpa*. Other groups were constituted by more than two members.

To find the possible explanation of the classification, MEME analysis was implemented to identify the conserved motifs in the protein sequences with default parameters and 1 to 15 motifs ranged from 5 to 50 amino acids. The mast of XML file was downloaded and TBtools was used to rebuild the motif maps. According to Fig 1B and S1 Fig, the 36 proteins were assigned to seven groups according to the conserved motifs and consistent with phylogenetic results. Ten proteins in the group I had nine conserved motifs except PtAPX2. Sub-localization analysis via different programs suggested that group I proteins were mainly located in cytosol (Fig 1C and S1 Table), this result resembled the previous experimental studies which reported that AtAPX2 and OsAPX2 were located in cytosol [18, 25, 52], respectively. The group II contained eight proteins which had ten conserved motifs with one exception of AtAPX5. Interestingly, all of these proteins had motif ten in this group, which was different from other groups. Among the eight proteins, AtAPX3 and OsAPX3 have been experimentally proved to locate in peroxisome [53, 54], respectively. Sub-localization analysis according to different programs, especially PANTHE, indicated that proteins in this group could be assigned to peroxisome (Fig 1C and S1 Table). The group III had eight motifs and contained only one protein, CreAPX-heme, which was located in mitochondria. The group IV had one unique motif nine compared with other groups in addition to several conserved motifs. Previous studies also investigated the sub-localization of OsAPX5, 6, 7 and 8 with different methods, the results showed that the former two proteins were located in mitochondria [54–56], and the latter two were distributed in chloroplast in rice plant [56]. Consequently, this group of proteins might be mainly located in mitochondria and chloroplast (Fig 1C and S1 Table). The rest three groups were V, VI and VII, sub-localization analysis indicated that all the proteins in the four groups mainly located in the chloroplast (Fig 1C and S1 Table). Studies on APXs localized in the chloroplast of *Chlamydomonas reinhardtii*, *Physcomitrella patens*, *Selaginella moellendorffii* and *Arabidopsis thaliana* suggested that there was a strong evolutionary pressure on maintaining the activity of the enzymes during plant evolution [57], this result resembled to the distribution of different conserved motifs in the groups III, VI, V and VII. Among the four groups, group V and VII contained four and two proteins, respectively. Both the proteins in the two groups had four to five conserved motifs. While there was only one protein containing eight motifs in group III and four motifs in group VI, respectively. Additionally, phylogenetic tree suggested that group VI and VII differed from group I to V, although both the two groups contained several conserved motifs, such as motif three, four and six. The reasonable explanation might be attributed to the motif fifteen which was only detected in the three proteins from group VI and VII. In general, the 36 APXs from the selected species showed high evolutionary conservation, indicating these enzymes played vital roles in plant growth and development.

### Modification analysis of APXs

**Fig 2:**
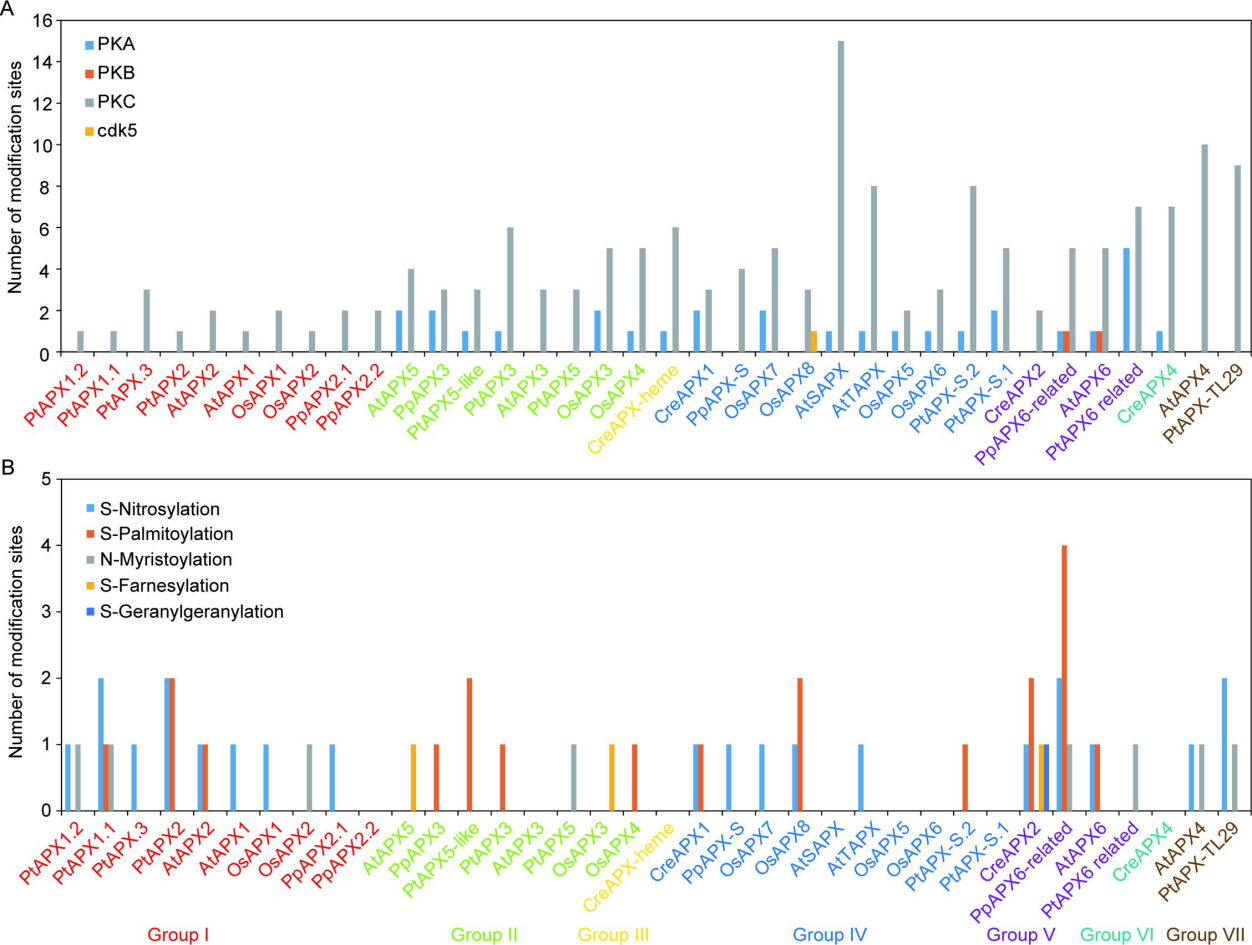
Modification analysis of the 36 APXs. A: Number of Phosphorylation sites. B: Number of S-Nitrosylation, S-Palmitoylation, N-Myristoylation, S-Farnesylation and S-Geranylgeranylation sites.

To analyze the possible modification sites, we submitted the APXs to several bioinformatics software to implement the prediction. Protein phosphorylation is a key regulatory post-translational modification involved in different cellular processes in plant cells [58]. The NetPhos software 3.1 was firstly used to predict the phosphorylation sites at serine, threonine and tyrosine where protein phosphorylation occurred mostly in eukaryotic. According to Fig 2A and S2 Table, all the 36 APXs contained higher number of PKC sites. Interestingly, APXs in group I and group VII contained only PKC site compared with other groups. Members in group II (except AtAPX3 and PtAPX5, which contained only PKC site), III and VI had PKA and PKC sites. There were three kind of phosphorylation sites in APXs of group IV, but only one enzyme contained cdk5 site, other enzymes had PKA and/or PKC site(s). In group V, PpAPX6-related and AtAPX6 contained PKA, PKB and PKC sites, PtAPX6 related had PKA and PKC sites, CreAPX2 contained only PKC sites. Among all the enzymes, AtSAPX contained fifteen phosphorylation sites at the given threshold, which might attribute to its localization and functional specificity. Previous studies have certified that OsAPX6 was able to be phosphorylated at GL^13^**s**AA and PP^172^**s**PA sites [59]. In this study, the prediction showed that GL^13^**s**AA was the PKA site, while PP^172^**s**PA was other unknown kinase site and not included in the Fig 2A. Therefore, these predictions were, to some extent, credible.

Studies on the pea leaves indicated that S-Nitrosylation was able to enhance cytosolic APX activity [34], which contrasted with the result that S-Nitrosylation inhibited the cytosolic APX activity during the PCD process in tobacco bright yellow-2 cells [60]. Despite the contradiction, the two studies revealed that S-Nitrosylation could indeed regulate the APXs activity. Therefore the S-Nitrosylation was predicted by GPS-SNO 1.0 software. According to the Fig 2B and S2 Table, 18 enzymes in group I, IV, V and VII contained the possible sites under the setting parameters, indicating S-Nitrosylation indeed played vital roles in modulating the activity of APXs in the selected species. Previous reports have shown that AtAPX1 activity could be enhanced by S-Nitrosylation and partially inhibited by denitrosylation to modulate root growth pattern with auxin regulation [61], indicating APXs not only functioned redox regulation, but also regulated plant growth and development. Surprisingly, no S-Nitrosylation site was observed in members of group II, III and VI. These results indicated that different groups of APXs might have slight unknown functions.

S-Palmitoylation, which is uniquely reversible among different protein modifications, has potential and rapid spatiotemporal regulation of protein functions [62]. This modification might involve in modulation of phosphorylation signaling cascades in plant species [63, 64]. Few reports focused on S-Palmitoylation of APXs to date. Therefore, S-Palmitoylation was analyzed according to the CSS-Palm 4.0. The result showed that 13 APXs in group I, II, IV and V containing S-Palmitoylation sites were observed, indicating the function of these enzymes might be modulated by the S-Palmitoylation.

N-Myristoylation is an irreversible protein modification and controls function of several proteins involved in plant development and redox balance [65]. However, few studies reported the relationship between N-Myristoylation and function of APXs. According to our result, 8 APXs in group I, II, V and VII contained N-Myristoylation sites. Subsequently, S-Farnesylation and S-Geranylgeranylation were also analyzed. S-Farnesylation, which plays important biological roles, is a covalent isoprenoid modification and able to increase the hydrophobicity of proteins to enhance their affinity for membranes [66]. S-Geranylgeranylation is another lipid modification in proteins. However, both the two modifications were seldom reported in the plant species. The result showed that 3 APXs in group II and V and 1 APX in group V contained the S-Farnesylation and S-Geranylgeranylation sites, respectively (Fig 2B). Interestingly, PpAPX6-related had up to four S-Palmitoylation sites (Fig 2B). The abovementioned enzyme modifications were only the results predicted by the software and should be confirmed by the future experiments, although several modifications have been stated in previous studies [61, 63, 64].

### Identification of cis-regulatory elements of APX promoters

**Fig 3:**
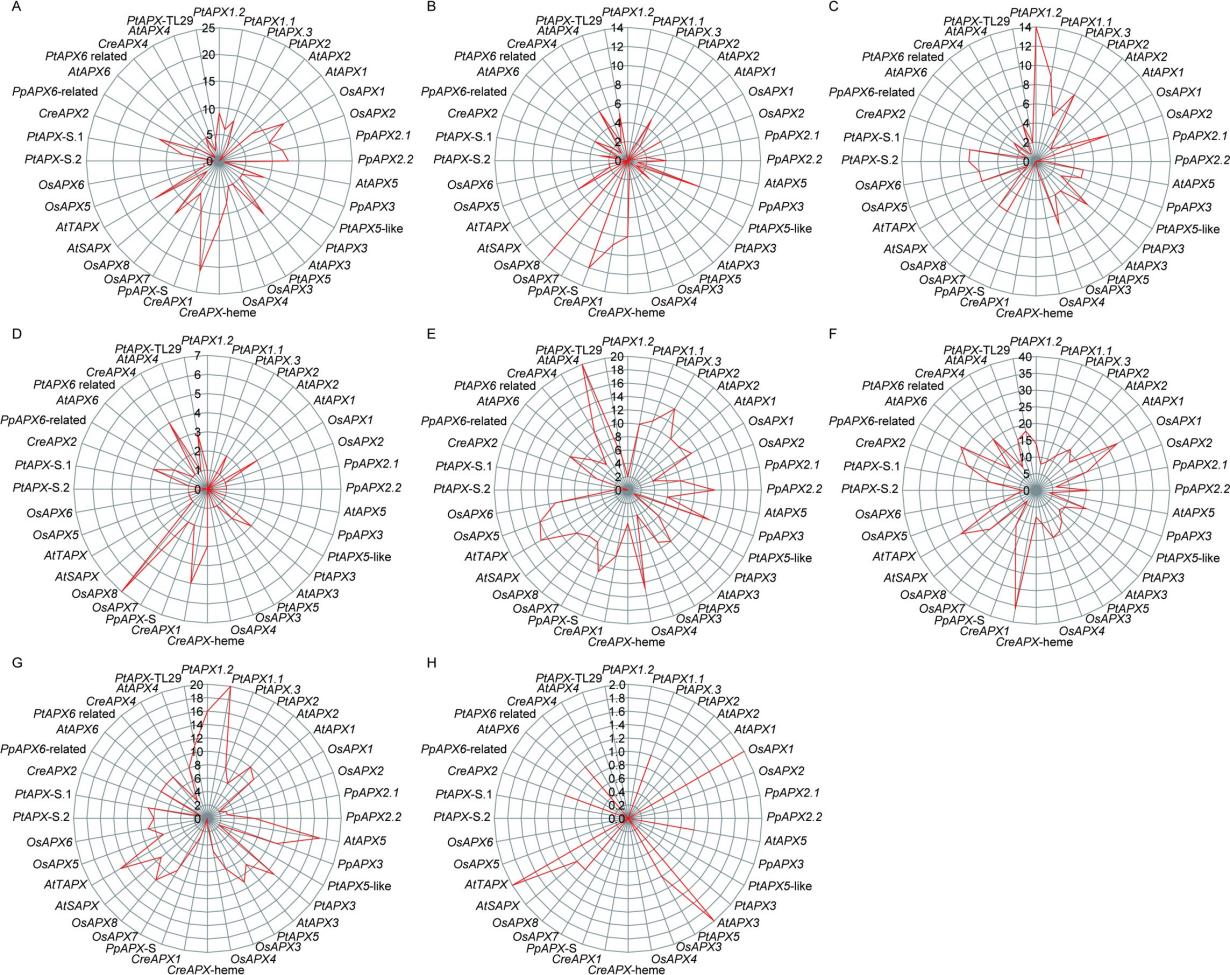
Conserved cis-regulatory elements in the promoters of 36 APXs. A: Response to ABA related elements including ACACNNG, ACGTG, ACGTGKC, ACGTSSSC, CAAACACC, CACGTGGC, CATGCCGCC, CCTACGTGGC, GCCGCGTGGC, MACGYGB, RYACGTGGYR, TACGTGTC and YACGTGGC. B: Response to dehydration related elements including ACCGAC, ACCGAGA, GTCGAC, RCCGAC and RYCGAC. C: Response to salt element (GAAAAA). D: Response to low temperature related elements including ACCGACA, CCGAAA and CCGAC. E: MYB elements including AGATCCAA, CNGTTR, CTAACCA, GTTAGGTT, GTTAGTT, TAACTG, WAACCA and YAACKG. F: MYC elements including CAACGTG, CACATG, CANNTG and CATGTG. G: POLLEN element involved in pollen and anther development (AGAAA). H: Axillary bud outgrowth related elements including AAACCCTA, CCACGTCA and GGCCCAWWW.

Cis-regulatory elements are key switches for the transcriptional modulation of a dynamic network of genes expression. During abiotic and biotic responses, hormone responses and plant development, different transcription factors interacted with cis-regulatory elements to determine transcription initiation events [67]. APX functions degradation of H_2_O_2_ which is involved in abiotic and biotic stresses. In order to explore the possible conserved elements related to stresses response and developmental regulation in the promoters of 36 APXs, PLACE software was used to predict the cis-regulatory elements. The results were shown in Fig 3 and S2 Table.

**Fig 4:**
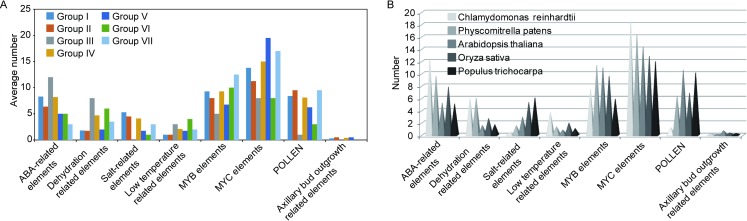
Number of elements in the seven groups and species. A: Average number of elements in the group I to VII. B: Average distribution of elements in the five species.

All the promoters of 36 APXs possessed the elements responding to ABA (Fig 3A). The promoter of *CreAPX1* assigned to group IV from *Chlamydomonas reinhardtii* was detected 21 ABA related elements and the most one among the investigated genes. In the same group, promoters of *PpAPX*-S in *Physcomitrella patens*, *AtTAPX* in *Arabidopsis thaliana* and *OsAPX8* in *Oryza sativa* individually contained 10, 14 and 13 ABA related elements. While promoters of *PpAPX2*.*1* and *PpAPX2*.*2* from *Physcomitrella patens* and *OsAPX1* and *OsAPX2* from *Oryza sativa* were detected 12, 13, 14 and 10 ABA related elements, respectively. All of the four APXs were members of group I. Promoter of *AtAPX3* from *Arabidopsis thaliana* in group II and *CreAPX*-heme from *Chlamydomonas reinhardtii* in group III contained 13 and 12 ABA related elements separately. Another gene, *CreAPX2*, from *Chlamydomonas reinhardtii* in Group V was found 12 ABA related elements in the promoter. According to Fig 4A and S2 Table, the average number of ABA related elements in genes of group III (12), I (8.3) and IV (8.2) were more than other groups. Interestingly, no gene contained more than ten ABA related elements were detected in *Populus trichocarpa*. According to these results, ABA might induce expression of APXs significantly.

Subsequently, stresses related elements involved in dehydration, salt and low temperature were analyzed in the genes of seven groups from the five species (Fig 3B, 3C and 3D). Promoters of *OsAPX8* from *Oryza sativa* and *PpAPX*-S from *Physcomitrella patens* in group IV contained 13 and 12 dehydration related elements, respectively (Fig 3B). While promoters of *PtAPX1*.*2* in group I from *Populus trichocarpa* had up to 14 salt related elements (Fig 3C). Other genes in each group involved in dehydration and salt stress had no more than ten elements, no genes in the seven groups contained more than ten low temperature related elements were observed in the five species (Fig 3D). The average number of dehydration related elements in group I to VII were 1.8, 1.75, 8, 4.7, 2, 6 and 3.5, that of salt related elements in group I to VII were 5.3, 4.5, 0, 4.1, 1.75, 1 and 3, that of low temperature related elements were 1, 1, 3, 2.1, 1.75 and 4,2 (Fig 4A).

Additionally, several other elements were also analyzed in the promoters of the 36 APXs. The results showed that nineteen and twenty-six genes contained more than ten MYB and MYC elements in the seven groups, respectively (Fig 3E and 3F). Group VII and V individually contained more MYB and MYC elements, compared with other groups (Fig 4A). Cis-element (POLLEN) involved in pollen and anther development was analyzed. According to Fig 3G, promoters of *PtAPX1*.*1* and *PtAPX1*.*2* in group I, *AtAPX5*, *PpAPX3*, *PtAPX3* and *PtAPX5* in group II, *OsAPX8* and *AtTAPX* in group IV and *PtAPX*-TL29 in group VII contained more than ten elements (Fig 3G). Among the seven groups, group II and VII contained more POLLEN elements than other groups (Fig 4A). Interestingly, seven genes assigned to group I, II, IV and V from four species contained one to two elements related to axillary bud outgrowth (Fig 3H). The average number of this element was less than other elements (Fig 4A).

To observe the average distribution of above-mentioned elements in each plant species, the eight types of element were summarized in Fig 4B and S2 Table. The results showed that APXs in *Chlamydomonas reinhardtii* and *Physcomitrella patens* contained more ABA related elements, compared to *Arabidopsis thaliana*, *Oryza sativa* and *Populus trichocarpa*. Among the three higher plants, APXs in *Oryza sativa* owned more ABA related elements. The similar trend was observed in dehydration related elements. Intriguingly, salt, POLLEN and axillary bud outgrowth related elements were abundant in higher plants, compared to *Chlamydomonas reinhardtii* and *Physcomitrella patens*. In addition, low temperature related elements in *Chlamydomonas reinhardtii* were less than other species. Meanwhile, similar trend was also observed in the MYB elements of *Chlamydomonas reinhardtii* and *Populus trichocarpa*.

According to the aforementioned results, APXs in different groups and species contained different numbers of cis-regulatory elements. These might be related with differences of APXs localizations and functions, and evolutionary status of species. Studies in the rice plants showed that ABA enhanced the expression of *OsAPX1* significantly [68].Mutation of cis-regulatory elements resulted in pleiotropic effects [69], such as changes in cis-regulatory element of *GRAIN WIDTH 7* (*GW7*) gene promoter produced slender grains [70]. Further studies illustrated that the cis-regulatory elements functioned via a combination rather than a single way to regulate the genes expression patterns to withstand different stresses [71]. Consequently, cis-regulatory elements were crucial for the plant development and resistance to stresses, especially elements in promoters of APXs, which played vital roles in ASH-GSH pathway.

### Three-dimensional models of eight rice APXs

**Fig 5:**
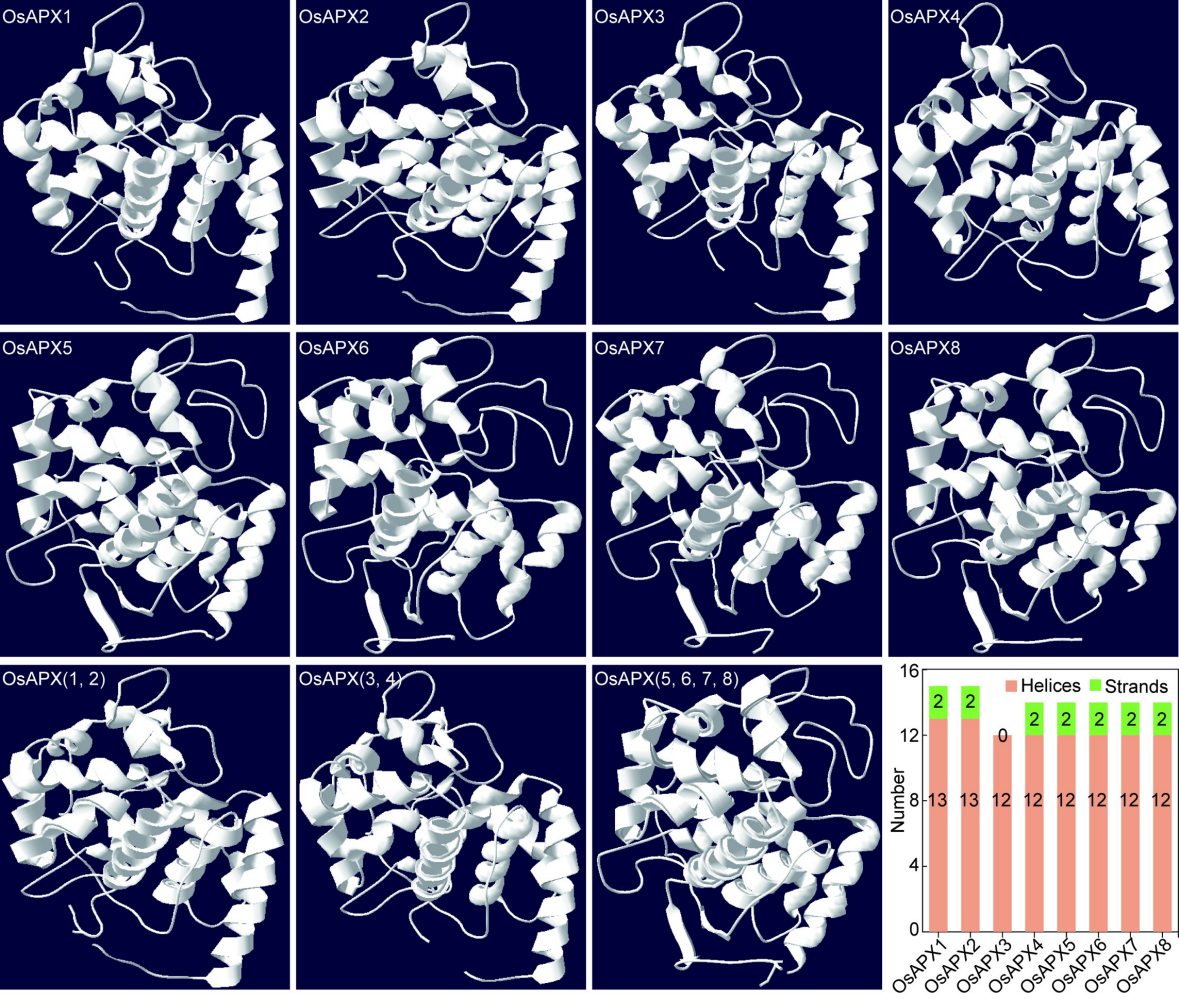
The three-dimensional models of eight rice APXs.

Since the protein sequences of APXs from different species are highly conserved and *Oryza sativa* is one of important food crop and model plant, subsequent analysis of the enzymes were carried out with rice APXs. Due to the importance of protein or enzyme structures to their functions, we firstly used the Swiss-Model software to construct the three-dimensional models of eight rice APXs. Fig 5 showed that the models of rice APXs were divided into two major groups according to the three-dimensional structures. One group contained OsAPX1, OsAPX2, OsAPX3 and OsAPX4, while the other group was OsAPX5, OsAPX6, OsAPX7 and OsAPX8. In order to explore the differences among members of the same group, we overlapped their three-dimensional models. Interestingly, when the same group members were overlapped, we found that the former group could be further divided into two sub-groups, one contained OsAPX1 and OsAPX2, the other contained OsAPX3 and OsAPX4. However, the members of later group could be overlapped conveniently. This result was similar with the eight enzymes localizations. Fig 5 also suggested that OsAPX1 and 2 contained 13 helices and the rest of APXs had 12 helices, all the APXs except OsAPX2 contained two strands. These results indicated that the eight enzymes contained similar helices and strands, which were consistent with their function that the enzymes mainly catalyzed H_2_O_2_ into H_2_O. Additionally, predicted model of OsAPX2 contained metal ligand (K) and OsAPX7 and 8 had HEM ligands (protoporphyrin ix containing Fe). APX belongs to the class I heme-peroxidases and should contain HEM ligands. However, only two predicted models contained HEM ligands, these might attributed to the PDB of the basis. Therefore, further analysis of APXs three-dimensional models in the rice should be conducted via experimental technologies. According to the abovementioned results, the rice APXs might function differently due to their three-dimensional structures and ligands, although this kind enzyme mainly maintained the H_2_O_2_-eliminating ability in the cells.

### Interaction network of rice APXs

**Fig 6:**
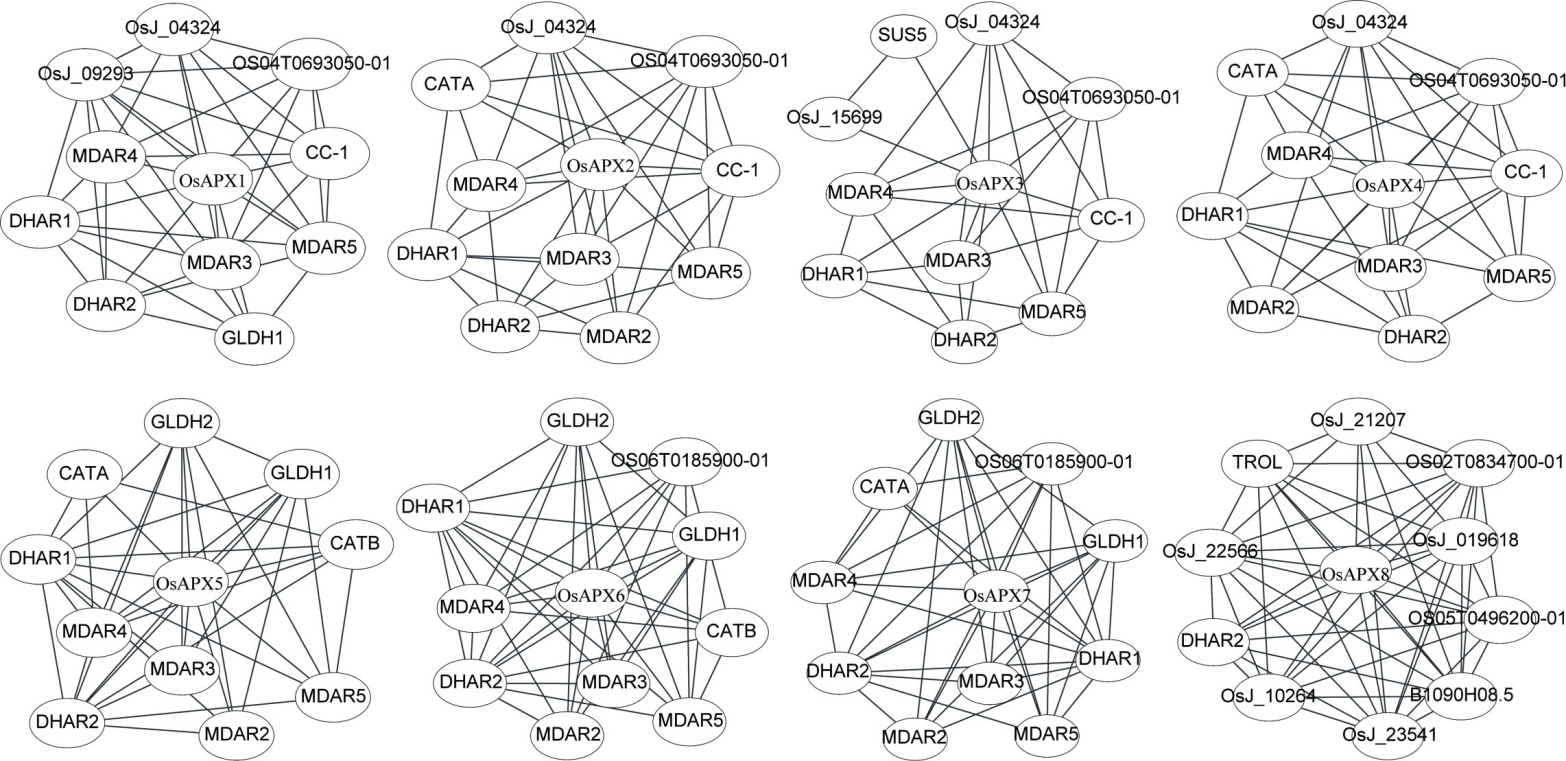
Predicted interaction partners of rice ascorbate peroxidase. DHAR1 and 2 (Dehydroascorbate reductase 1 and 2); MDAR2, 3, 4 and 5 (Monodehydroascorbate reductase 2, 3, 4 and 5); OS04T0693050-01, OsJ_04324 and CC-1 (Cytochrome c); GDLH1 and 2 (L-Galactono-1, 4-lactone dehydrogenase 1 and 2); CATA (Catalase isozyme A); CATB (Catalase isozyme B); Os06T0185900-01 (Glutathione peroxidase); OsJ_21207 (Putative bundle sheath defective protein); TROL (Thylakoid rhodanese-like protein); OS02T0834700-01 (Cell division inhibitor); OsJ_22566 (Thylakoid lumenal 16.5 kDa protein); OsJ_019618 (Peroxiredoxin Q); OS05T0496200-01 (Phosphoglycerate kinase); OsJ_10264 (Fructose-1,6-bisphosphatase); B1090H08.5 (Peptidyl-prolyl cis-trans isomerase); OsJ_23541 (Putative mRNA binding protein).

To analyze the potential interaction partners of rice APXs, networks were constructed using cytoscape software with STRING data (S2 Table). According to Fig 6, DHAR2 (Dehydroascorbate reductase 2) and DHAR1 were the mutual interaction partner of the eight and seven APXs from rice plant, respectively. The enzymes function as GSH-dependent dehydroascorbate reductase and play a vital role in plant cell growth by regulating content of ascorbate [72]. MDAR2 (Monodehydroascorbate reductase), MDAR3, MDAR4 and MDAR5 catalyze the conversion of monodehydroascorbate to ascorbate using NAD(P)H in this process [73, 74]. Among the four MDARs, MDAR3-5 were able to interact with OsAPX1, OsAPX2, OsAPX3, OsAPX4, OsAPX5, OsAPX6 and OsAPX7, while MDAR2 were the interaction partner of OsAPX2, OsAPX4, OsAPX5, OsAPX6 and OsAPX7. These results agreed with the fact that DHAR and MDAR are important components in the ascorbate-glutathione cycle to regeneration of ascorbate [75]. Previous studies showed that activity of APX and content of ascorbate were significantly decreased in the rice plants during salt stress condition [76], indicating the important relationship between APX and ascorbate. Additionally, OS04T0693050-01, OsJ_04324 and CC-1 were cytochrome c, functioning as electron carrier protein [77], and the mutual interaction partners of OsAPX1, OsAPX2, OsAPX3 and OsAPX4, which showed similar three-dimensional structures predicted by Swiss-Model analysis. GLDH (L-Galactono-1, 4-lactone dehydrogenase) which catalyzes the last step in the main pathway of L-ascorbic acid biosynthesis in higher plants plays vital roles in the cell developmental processes [78], this enzyme uses cytochrome c as electron acceptor to convert L-galactono-1, 4-lactone to L-ascorbic acid on the inner mitochondrial membrane [79]. Fig 6 suggested that GLDH1 and GLDH2 could interact with OsAPX5 and OsAPX6 located in the mitochondria. Interestingly, OsAPX7 located in the chloroplast was also the interaction partner of the two enzymes, OsAPX1 located in the cytosol could interact with GLDH1. These results indicated that GLDH played important roles in APXs function via regulating L-ascorbic acid balance. CATA and CATB were the catalase isozyme A and B, respectively. Both the two enzymes function to scavenge H_2_O_2_ together with APXs to regulate redox balance. CATA was located in the cytosol and CATB in the peroxisome [80]. CATA was interacted with OsAPX2, OsAPX4, OsAPX5 and OsAPX7 located in the cytosol, peroxisome, mitochondria and chloroplast, respectively, while CTAB was interacted with OsAPX5 and OsAPX6 located in the mitochondria (Fig 6). The detail mechanism of different localized protein interaction was still unknown. We also found that Os06T0185900-01 could interact with OsAPX6 and OsAPX7 (Fig 6). It was a glutathione peroxidase and essential for *in vitro* rice regeneration and redox homeostasis [81]. Surprisingly, the interaction partners (except DHAR2) of OsAPX8 differed to other APXs, indicating this enzyme might have different or special functions (Fig 6).

### Transcriptional profiles of APXs in rice

**Fig 7:**
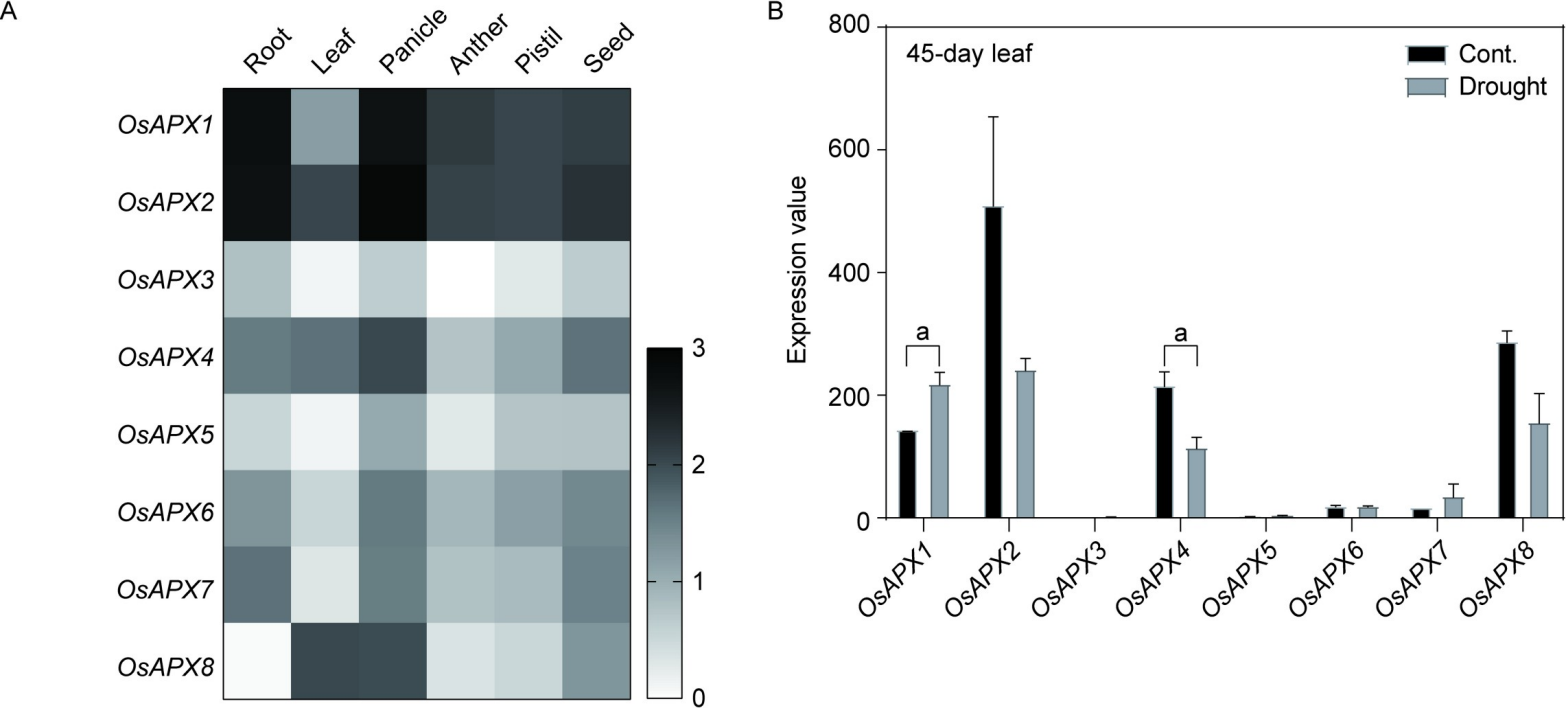
**The expression profiles of rice APXs in different organs or tissues (A) and under drought condition (B).** Two-tailed student *t* test, a: *p* < 0.05.

According to the phylogenetic analysis, we found that the APXs showed high evolutionary conservation, indicating these enzymes were important during plant growth and adaption to the environment. Since expression of genes is one crucial step in achieving their functions, we investigated the transcriptional profiles of eight rice APXs in different organs or tissues. According to Fig 7A and S2 Table, two cytoplasmic-located enzymes, OsAPX1 and OsAPX2, exhibited high expression trend in the investigated tissues or organs compared with other APXs in rice, especially in panicle. These result indicated that cytosolic APXs might play key roles in rice plant growth, development and reproduction. The single gene silence of the two enzymes have been certified their importance, although double genes mutants showed normal phenotypes [17, 18, 27]. However, the mechanisms were still unclear. Further analysis should be conducted via genetic, molecular and morphological methods to explore the functions of APXs. Fig 7A also indicated that the expression patterns of the eight APXs altered significantly in different rice organs or tissues.

Subsequently, the transcriptional profiles of rice APXs in different stresses were analyzed. Water is an important factor in agricultural production, drought stress severely impairs rice yield [82]. When 45-day rice plants were treated with drought stress, *OsAPX1* and *OsAPX4* were significant up-regulated and down-regulated in the leaf of rice plant, respectively (Fig 7B and S2 Table). No significant changes were observed in other APXs. Maruyama et al (2014) reported that more than 5000 and 6000 genes were up-regulated and down-regulated in the two-week old rice seedlings with three-day dehydration treatment [83], respectively. Among the differential expression genes, *OsAPX2*, *OsAPX4* and *OsAPX8* showed significantly down-regulated, while no changes were observed in other APXs genes under the treatment condition. Our result of *OsAPX4* expression changes was similar with previous study [83].

**Fig 8:**
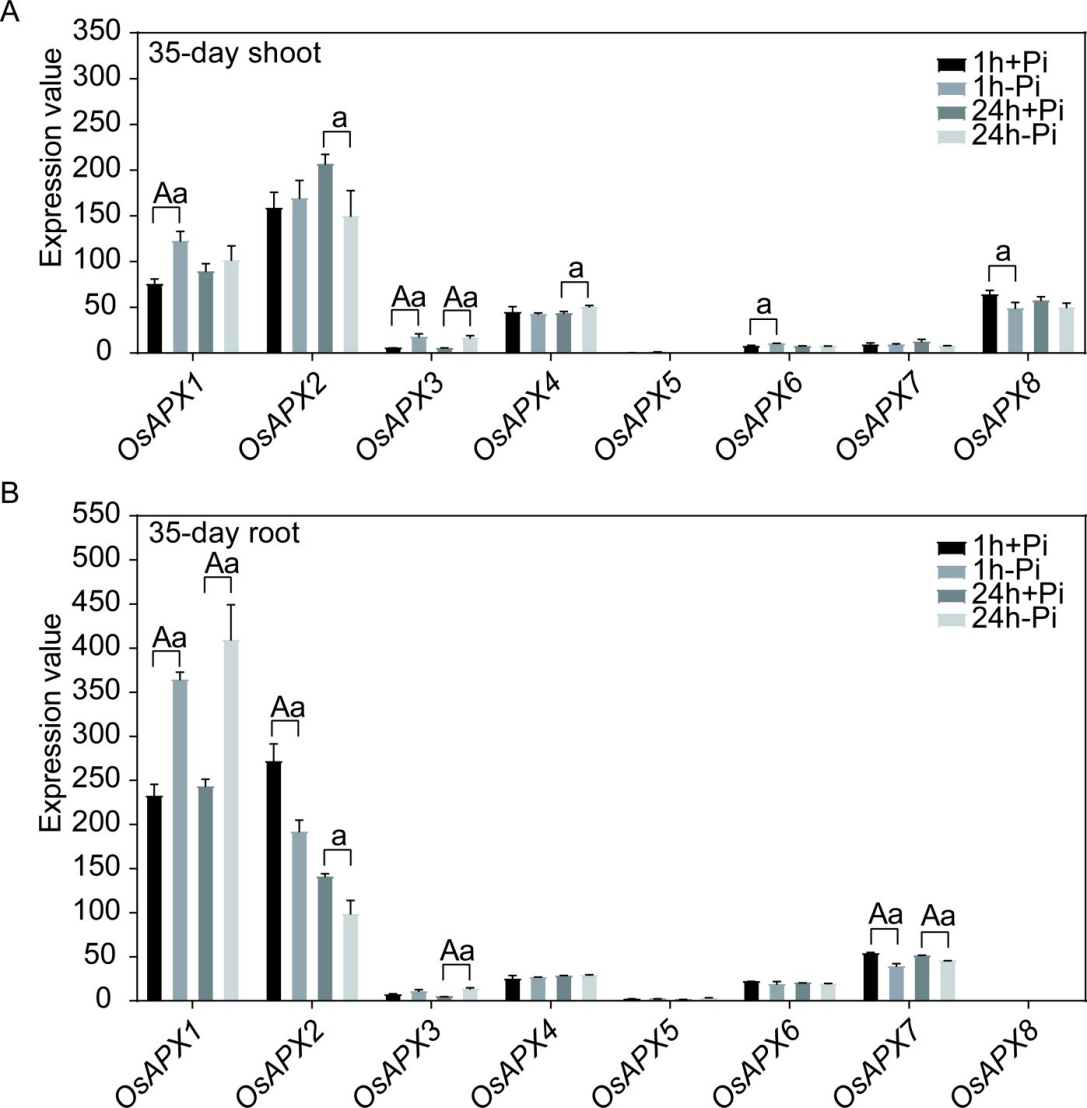
The expression profiles of rice APXs under Pi-free treatments. A: Expression profiles of APXs in 35-day shoot. B: Expression profiles of APXs in 35-day root. Two-tailed student *t* test, a: *p* < 0.05, A: *p* < 0.01.

Phosphorus (P) is a critical element for plant growth and productivity. Phosphate (Pi) is one inorganic bioavailable form of phosphorus and only less than 20% is available for plants [84]. For 67% of the world’s cultivable soils, Pi is a limiting factor [85]. When the 35-day old rice plants were cultivated in the Pi-free nutrient for 1 h or 24 h, *OsAPX1*, *OsAPX3*, *OsAPX4* and *OsAPX6* were up-regulated in the shoot (Fig 8A and S2 Table), while *OsAPX1* and *OsAPX3* were also exhibited up-regulation in the root (Fig 8B and S2 Table). These results were certified by previous reports that *OsAPX1* was able to up-regulated in the shoot and root of two-week old seedlings with twenty-two-day treatment without Pi [86]. In addition, *OsAPX2* in both shoot and root, *OsAPX8* in shoot and *OsAPX7* in root were significantly down-regulated after 1 h or/and 24 h treatment without Pi (Fig 8). The abovementioned results suggested that the expression profiles could be altered by short term Pi starvation, compared with long term treatment [86]. Further analysis indicated that rice APXs with same sub-localizations exhibited no changes or similar expression trends (in shoot or/and root). However, cytoplasmic-located APXs did not follow this trend, the two enzymes presented opposite expression patterns especially in the root tissue (Fig 8). It suggested that the two APXs might possess potential functional differences in response to Pi-free stress.

**Fig 9:**
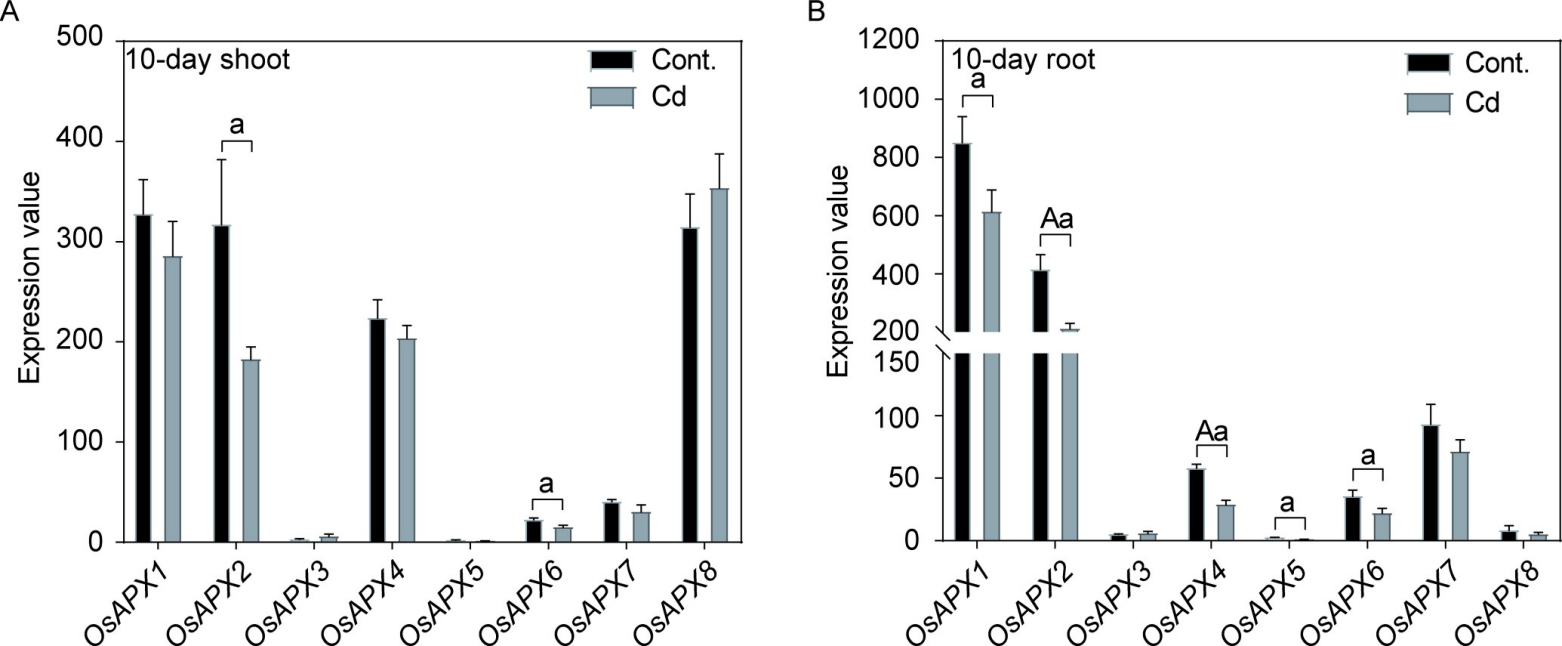
The expression profiles of rice APXs under Cd stress. A: Expression profiles of APXs in 10-day shoot. B: Expression profiles of APXs in 10-day root. Two-tailed student *t* test, a: *p* < 0.05, A: *p* < 0.01.

Cadmium (Cd) is toxic heavy metal and able to cause phytotoxicity and human disease [87]. Rice is the most important source of Cd due to its stable food supply for people consuming. Cytosolic APXs could protect chloroplast from oxidative stress [19]. Therefore, exploring Cd stress on rice plants is of great importance for controlling Cd content. When 10-day old rice seedlings were treated with Cd stress, the expressions of *OsAPX2* and *OsAPX6* were significantly decreased in rice shoot, while other APXs showed no differences, compared with controls (Fig 9A and S2 Table). However, five APXs showed obvious down-regulated in the 10-day old rice root (Fig 9B and S2 Table). These results illustrated that Cd stress had a great influence on expressions of rice APXs in 10-day old root. Totally, Cd stress significant altered expression profiles of rice APXs.

**Fig 10:**
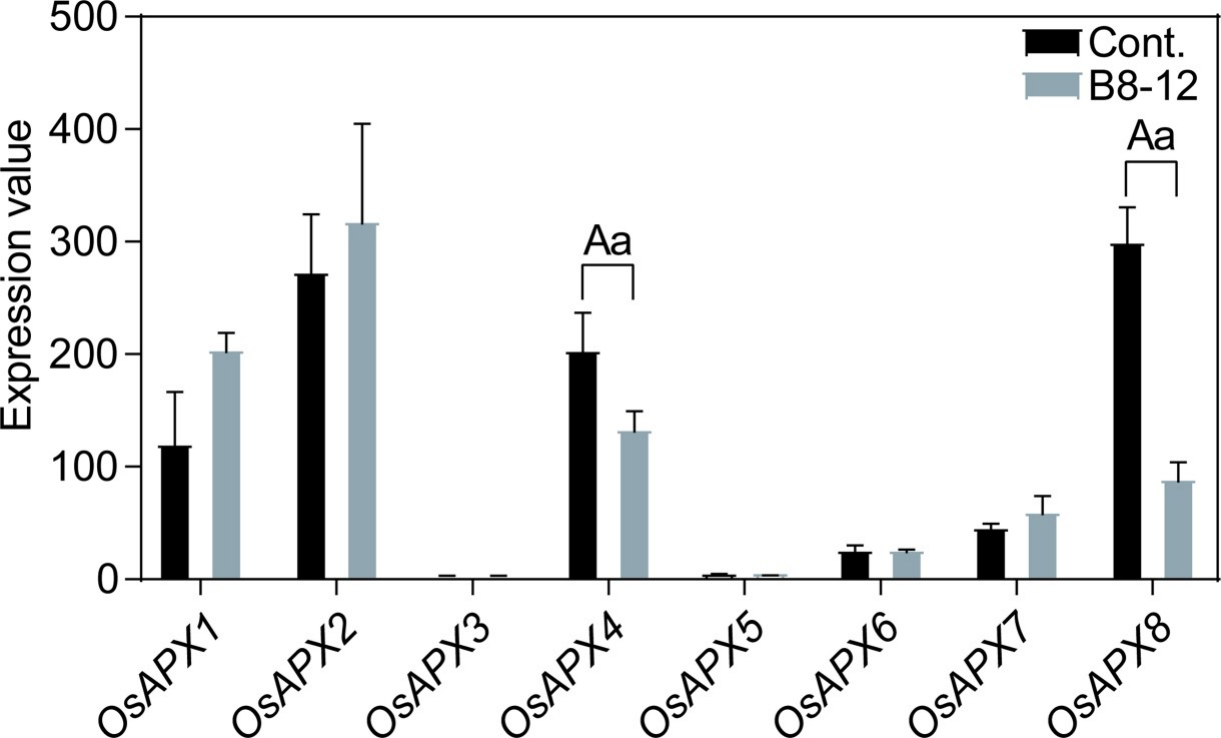
The expression patterns of rice APXs in 16-day leaf with *Xanthomonas oryzae pv*. *oryzicola* B8-12 infection for 48 h. Two-tailed student *t* test, a: *p* < 0.05, A: *p* < 0.01.

Finally, we analyzed the expression profiles of the eight rice APXs in the 16-day old leaf with *Xanthomonas oryzae pv*. *oryzicola* B8-12 infection for 10 days. The *Xanthomonas oryzae pv*. *oryzicola* could cause rice plants to infect bacterial leaf streak [88]. According to Fig 10 and S2 Table, only *OsAPX4* and *OsAPX8* exhibited different expression patterns compared with control group, indicating other APXs were insensitive to *Xanthomonas oryzae pv*. *oryzicola* B8-12 bacteria.

## Conclusion

In the present study, APXs from different plant species were analyzed and showed high evolutionary conservation. The 36 APXs from *Chlamydomonas reinhardtii*, *Physcomitrella patens*, *Arabidopsis thaliana*, *Oryza sativa* and *Populus trichocarpa* could be divided into seven groups. The classifications were consistent with sub-localization of APXs. Further analysis suggested that the APXs contained abundant phosphorylation sites. APXs in group I and VII contained only PKC site. Promoters of the selected APXs genes contained abundant ABA, MYB and MYC elements. The average number of elements in different groups altered significantly. All the rice APXs were able to interact with dehydroascorbate reductase 2, and expressed differently in different rice tissues or organs, especially cytosol-located OsAPX1 and OsAPX2. When the rice plants were treated with the abiotic and biotic stresses, the rice APXs showed different expression profiles to maintain normal physiological activities. Under drought condition, *OsAPX2* and *OsAPX4* were significantly up- and down-regulated, respectively. Under Pi-free condition, *OsAPX3* in shoot and *OsAPX1* in root showed significant up-regulation, while *OsAPX2* and *OsAPX7* were significantly down-regulated in the root. Interestingly, *OsAPX2* and *OsAPX6* showed significant down-regulation in the shoot and root under Cd condition, meanwhile *OsAPX1* and *OsAPX6* in the root were also down-regulated. When the rice plant was subjected to biotic stress such as *Xanthomonas oryzae pv*. *oryzicola* B8-12 infection, *OsAPX4* and *OsAPX8* exhibited significant down-regulation. The present investigation laid a foundation for further functional exploration and application of APXs.

## Supporting information

S1 Fig The conserved sequences of motif 1 to 15. (TIF)

S1 Text APXs sequences used in this paper. (TXT)

S1 Table Localization of the 36 APXs from different species searched from databases and analyzed by CELLO and PSORT. (XLSX)

S2 Table Data used in this paper. (XLSX)
