# Supplementary figures and images for: Comparative analysis of ascorbate peroxidases (APXs) from selected plants with a special focus on Oryza sativa employing public databases
Source: PLoS One. 2019 Dec 19;14(12):e0226543. doi: 10.1371/journal.pone.0226543 (PMC6922425; doi:10.1371/journal.pone.0226543)

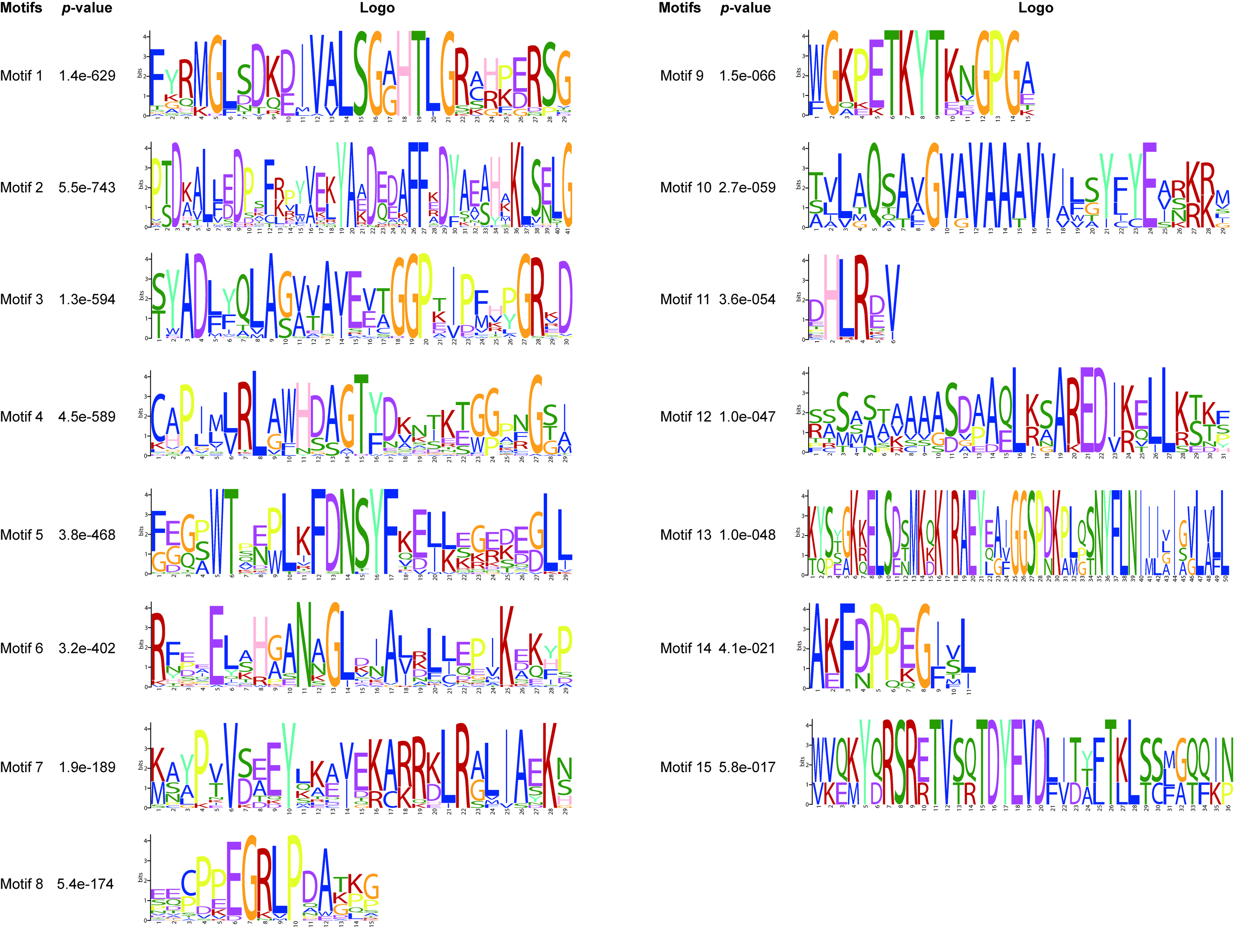

Supplement: S1 Fig — (TIF) [file pone.0226543.s001.tif]
